# Acute kidney injury after cardiac surgery is associated with mid-term but not long-term mortality: A cohort-based study

**DOI:** 10.1371/journal.pone.0181158

**Published:** 2017-07-10

**Authors:** Alejandro Ferreiro, Raúl Lombardi

**Affiliations:** 1 Centro de Nefrología, Universidad de la República, Montevideo, Uruguay; 2 Instituto Nacional de Cirugía Cardíaca, Montevideo, Uruguay; 3 Departamento de Medicina Crítica, Servicio Médico Integral, Montevideo, Uruguay; University of Sao Paulo Medical School, BRAZIL

## Abstract

Acute kidney injury (AKI) in cardiac surgery is associated with complications, early and late mortality and increased health care expenditures. The overall dynamic comorbidity-adjusted contributions of an episode of AKI on mortality during long-term follow-up have not been fully explored. A longitudinal cohort of 7075 adult patients admitted for cardiac surgery were enrolled in the study. Follow-up data were obtained through telephonic survey after 1, 5, 10, and 15 years or from the National Mortality Registry. All-cause mortality was assessed at five time intervals: I) 30 days after surgery to 1 year; II) 1 to 3 years; III) 3 to 5 years; IV) 5 to 10 years; and V) 10 to 15 years. For the adjustment of mortality for comorbidity and pre-, intra- and postoperative variables, Cox proportional hazard regression models were conducted within each period. The overall incidence of AKI was 36.1%. AKI was an independent predictor of death only during the first five years after surgery (30 days to 1 year: HR 1.834, 95% CI 1.459 to 2.306; 1 to 3 years: HR 1.285, 95% CI 1.023 to 1.610; and 3 to five years: HR 1.330, 95% CI 1.123 to 1.750). Only age, diabetes mellitus and CHF were associated with increased risk of death over the entire follow-up period. Our study demonstrates a transient association of AKI with long-term mortality that progressively decreases and vanishes five years after surgery. The knowledge of this dynamic is crucial to understanding this complex association, planning health care and allocating resources.

## Introduction

The relevance of acute kidney injury (AKI) as a major problem in hospitalized patients is unquestionable given its association with serious complications, early and late mortality [1,2], and increases in health care expenditures [3,4]. On the other hand, being acute kidney injury a potentially avoidable condition it raises the challenge for physicians and the health care system to identify risk factors in order to design effective preventive strategies. Lastly, growing attention has been devoted to the effect of AKI on adverse long-term outcomes, such as chronic kidney disease (CKD), cardiovascular disease and lower life expectancy [5], moving the classical individual and hospital-based dimension of AKI toward a public health dimension due to the impact of AKI on the global burden of disease [6]. The underlying mechanism by which AKI is associated with late untoward outcomes is still unclear. It was proposed that AKI *per se* does not lead to the aforementioned outcomes being just a marker of the burden of comorbidities. Otherwise, AKI could trigger a complex and still not well-understood mechanism by which permanent and progressive damage occur.

Approximately 1 million people per year are admitted for coronary artery bypass grafting around the world [7]. Among them, up to 30% will develop AKI [8], resulting in death for 10% to 60% depending on the severity of AKI [9,10]. Previous reports have fully described the risk patterns for AKI after cardiac surgery and have resulted in a variety of strategies aimed at preventing the occurrence of this ominous complication [11]. Recently, studies performed in a number of different circumstances including cardiothoracic surgery, have focused on long-term endpoints as renal function and mortality using lengths of follow-up ranging from 3 months to 10 years [12,13]. Usually, mortality at the end of follow-up was considered the final endpoint, thus reflecting total mortality accumulated across the period. To our knowledge, little attention has been paid to evaluating if the contribution of an episode of AKI to late mortality persists during all long term follow-up or if it declines during the aforementioned time period. We therefore carried out the present study with the aim of evaluating the net attributable impact of AKI after cardiac surgery on very long-term mortality and to characterize the risk profile as well as eventual changes in its pattern over time.

## Methods

### Design and setting

This is an observational, retrospective analysis of a longitudinal cohort of patients admitted for cardiac surgery. The prospectively collected database of the *Instituto Nacional de Cirugia Cardiaca* (INCC, Montevideo) located in the *Servicio Medico Integral* previously described was used for the analysis [14]. In brief, the database includes more than 750 variables reflecting demographics, comorbidities, previous cardiac history, preoperative left ventricular function, medical treatment pre-surgery, baseline renal function, type of procedure, procedural urgency, intraoperative and postoperative variables, perioperative support, complications, 30 day-mortality and medication at discharge.

Cardiac surgery in Uruguay is universally covered, and patients are referred from hospitals across the country to five reference hospitals, one of which is our Institute. Between January 1, 2000 and December 31, 2013, 9778 patients received surgery in our hospital, representing 38.8% of the total 25,216 patients undergoing cardiac surgery nationwide. Uruguay’s uniform access to the health care system minimizes selection bias. As a result, our database broadly represents the adult Uruguayan population that underwent cardiac surgery during this time period. The accessibility to the health system has not undergone modifications, as there were no major changes in the surgical technique in the study period. Follow-up data were obtained through systematic telephonic surveys after 1, 5, 10, and 15 years using a standardized form designed for this purpose. Alternatively, when information on date and cause of death was not available, the data were obtained by cross-referencing the INCC database with the National Mortality Registry.

### Study period and inclusion criteria

All consecutive adult patients (>18 years old) receiving cardiac surgery between January 1, 2000 and December 31, 2013 were enrolled in the study. Inclusion of patients was concluded in December 2013 in order to have at least one year of follow-up data. To avoid the confounding impact of hospital mortality on long-term survival analysis, only patients still living 30 days after surgery were included in the analysis.

### Exclusion criteria

Patients with CKD stage 5, dialysis-dependent end stage renal disease and previous kidney transplantation were excluded. Patients who received ascending aorta surgery were also excluded given that the high mortality rate associated with this surgery may bias the results.

### Definitions

AKI was defined and categorized according to KDIGO creatinine-based definition criteria [15]. Serum creatinine at anesthesia induction was considered as reference serum creatinine, and the peak value was the highest level of serum creatinine during hospitalization within the time frame according to KDIGO criteria. The definition and categorization of CKD was adopted from the KDIGO glomerular filtration criteria proposed in 2012 [16]. Baseline renal function was assessed by the CKD-EPI formula [17].

### Covariates selected for the analysis

1. Preoperative: age, gender, BMI, smoking, COPD, hypertension, diabetes (requiring and not requiring insulin), peripheral vascular disease, cerebrovascular disease, CKD stage, cardiovascular antecedents (other) prior myocardial infarction, LVEF, prior heart surgery, preoperative condition (critically ill/not critically ill). Logistic EuroSCORE were calculated for all patients. Age was considered as the patient’s age at the beginning of each period.

2. Surgery: surgery type (CABG, valve, combined) and surgical priority (emergency/elective surgery). Cardiopulmonary by-pass time, cross-clamp time and lowest hematocrit were not considered due to the high number of off-pump CACG surgeries performed in our Institute, which biases the logistic regression analysis due to the resulting large amount of missing data.

3. Postoperative period: AKI as dichotomous variable (yes/no), maximum KDIGO stages, stroke, pneumonia and deep sternal wound infection.

Additionally, demographics, type of cardiac surgery, and baseline renal function were used for comorbidity risk adjustment. Logistic EuroSCORE was not considered in order to avoid redundancy because its single components were already included in the analysis.

### Outcomes

All-cause mortality adjusted for pre, intra and postoperative variables were assessed at five time intervals through the end of follow-up: I) from 30 days after surgery to 1 year; II) 1 to 3 years; III) 3 to 5 years; IV) 5 to 10 years; and V) 10 to 15 years according to the follow-up schedule of the *Insituto Nacional de Cirugía Cardíaca*. For the time-stratified analysis, patients who died in each period were excluded from the analysis for the following period.

### Ethical considerations

Confidentiality was protected through the de-identification of personal data. Informed consent was waived due to the observational nature of the study. The study was approved by the Ethics Committee of the Hospital de Clínicas, Universidad de la República.

### Statistical analysis

Data are presented as mean and standard deviation (SD) or median and interquartile range (IQR) according to distribution and type of data. Categorical variables were expressed as proportion. Chi-squared (for categorical variables), t-tests, ANOVA, Mann-Whitney *U* tests, Kruskal-Wallis tests or Wilcoxon rank sum tests (for continuous variables) were used for bivariate comparison between groups. Time-survival estimation curves were generated using Kaplan-Meier estimates, and the differences between survival curves were evaluated by log rank test. For the identification of factors associated with mortality, a Cox proportional hazard regression model within each time period was used. Log-rank test pooled over strata or Breslow test were used for comparing the equality of survival distributions for the different levels of the factors. All variables significant at the *p* <0.1 level in bivariate analysis were entered into the multivariate model for each of the 5 determined periods (forward stepwise Cox regression) provided they were present in at least 2% of the sample. In the case of age, which was a continuous variable where the relationship with outcome was not linear, the age at the beginning of each period was considered in the analyses. Significant variables were entered into the model one at a time, beginning with the variable having the lowest *p* value. Variables that significantly improved the fit of the model were retained and forced into subsequent models. Stability of the model was checked every time a variable was entered. The final step was to search for first-degree interaction. The criteria for including an interaction term were that it had to be significant at *p value* <0.05, 1% of the sample had to exhibit that combination of factors and the combination had to be clinically relevant. Once the final model was developed (evaluated trough the -2 log-likelihood omnibus test and the Wald test), the weights attributed to each variable after adjustment for co-variables were obtained from the Cox regression *β* estimated coefficients (hazard ratios and 95% CI). Only the final model is reported here. The IBM statistical package SPSS Statistics version 22 was used for data processing and statistical analyses.

## Results

Seven thousand seventy-five patients were included in the analysis. Demographic and clinical characteristics of the population are presented in Table 1. The overall incidence of AKI was 36.1% (2554 of 7075), of which 75.9% was KDIGO stage 1, 16.4% was KDIGO stage 2 and 7.7% was KDIGO stage 3. AKI was associated with older age, comorbidities, baseline renal function, EuroSCORE, previous cardiac surgery, non-coronary surgery and post-operative complications. Additionally, AKI patients had worse short term outcomes and longer MV time and hospital-LOS. Table 2 displays the number of patients, frequency of AKI and the corresponding KDIGO stages in each of the five study periods. 1986 patients (28.1%) completed the maximum follow-up time of 15 years. The remaining patients were censored in each period due to death or reaching the maximum follow-up time (from surgery to December 31, 2014).

**Table 1:** Demographic and clinical characteristics of 7075 patients by AKI stage allocation.

				AKI			
	No AKI 4521 63.9%	All AKI 2554 36.1%	*P*	KDIGO 1 1938 75.9%	KDIGO 2 418 16.4%	KDIGO 3 198 7.7%	*P*
Age, years mean (SD), range	63.8(11.1), 18–88	67.7 (9.8), 19–91	<0.001	67.4 (9.8), 19–91	68.8 (9.5), 32–88	68.3 (10.3), 24–87	<0.001
Age >80 years n,%	202 (4.5)	205 (8.0)	<0.001	137 (7.1)	47 (11.2)	21 (10.6)	<0.001
Female sex n,(%)	1497 (33.1)	879 (34.4)	0.14	641 (33.1)	172 (41.1)	66 (33.3)	0.010
Diabetes mellitus n,(%)	1113 (24.6)	763 (29.9)	<0.001	551 (28.4)	145 (34.7)	67 (33.8)	<0.001
Smoker n,(%)	1264 (28.0)	610 (23.9)	<0.001	477 (24.6)	90 (21.5)	43 (21.7)	0.001
COPD n,(%)	291 (6.4)	181 (7.1)	0.29	136 (7.9)	31 (7.4)	14 (7.1)	0.75
SCr mg/dL, mean (SD)	1.07 (0.34)	1.09 (0.46)	<0.001	1.08 (0.35)	0.95 (0.23)	1.47 (1.07)	<0.001
eGFR ml/m/m^2^ BS mean (SD)	71.3 (19.9)	70.0 (22.2)	<0.001	69.6 (20.5)	75.5 (21.4)	62.2 (33.6)	<0.001
PVD n,(%)	290 (6.4)	251 (9.8)	<0.001	189 (9.8)	40 (9.6)	222 (11.1)	<0.001
CVD n,(%)	88 (1.9)	56 (2.2)	0.27	35 (1.8)	12 (2.9)	9 (4.5)	0.038
Hypertension n,(%)	3089 (69.8)	1897 (75.0)	<0.001	1439 (75.0)	312 (75.2)	146 (74.9)	<0.001
Previous MI n,(%)	1276 (28.2)	680 (26.6)	0.078	515 (26.6)	117 (28.0)	48 (24.2)	0.37
LVEF 30–50% n,(%)	1544 (60.5)	935 (36.6)	0.007	713 (36.8)	140 (33.5)	82 (41.4)	0.016
LVEF <30% n,(%)	1544 (60.5)	75 (21.9)	0.007	53 (2.7)	14 (3.3)	8 (4.0)	0.016
Past cardiac surgery n,(%)	41 (0.9)	44 (1.7)	0.002	27 (1.4)	10 (2.4)	7 (3.5)	<0.001
Severity at CS n,(%)	136 (3.0)	101 (4.0)	0.021	67 (3.5)	16 (3.8)	(9.1)	<0.001
Logistic EuroSCORE mean (SD)	4.3 (4.6)	5.8 (6.0)	<0.001	5.5 (5.4)	6.1 (6.6)	(9.4)	<0.001
Non-CABG n,(%)	1683 (37.2)	1203 (47.1)	<0.001	897 (46.3)	207 (49.5)	99 (50.0)	<0.001
Isolated CABG n,(%)	2838 (62.8)	1351 (52.9)	<0.001	1041 (53.7)	211 (50.5)	99 (50.0)	<0.001
On-pump n,(%)	1747 (61.5)	981 (72.7)	<0.001	746 (71.7)	158 (75.2)	78 (78.8)	<0.001
Off-pump n,(%)	1091 (38.5)	368 (27.3)	<0.001	295 (28.3)	53 (24.8)	21 (21.2)	<0.001
MV time, hours mean (D)	9.9 (1.8)	22.5 (82.2)	<0.001	17.2 (50.0)	27.5 (105.8)	67.3 (198.8)	<0.001
Hospital-LOS, days mean (SD)	8.0 (7.0)	10.3 (10.0)	<0.001	9.3 (8.7)	11.4 (10.3)	18.1 (16.0)	<0.001
Postoperative stroke n,(%)	50 (1.1)	67 (2.6)	<0.001	43 (2.2)	13 (3.1)	11 (5.6)	<0.001
Postoperative MI n,(%)	32 (0.7)	38 (1.5)	0.001	31 (1.6)	4 (1.0)	3 (1.5)	0.009
Deep wound infection n,(%)	21 (0.5)	18 (0.7)	0.13	6 (0.3)	5 (1.2)	7 (3.5)	<0.001

COPD: chronic obstructive pulmonary disease; SCr: serum creatinine; eGFR: estimated glomerular filtration rate; PVD: peripheral vascular disease; CVD: cerebrovascular disease; LVEF: left ventricle ejection fraction; MI: myocardial infarction; CS: cardiac surgery; CABG: coronary artery by-pass grafting; MV: mechanical ventilation.

**Table 2:** Frequency of AKI and KDIGO stages in each study period.

	Patients* (n,%)	No AKI (n,%)	AKI (n,%)	KDIGO 1 (n,%)	KDIGO 2 (n,%)	KDIGO 3 (n,%)
30d-1 years	6956	4428 (63.6%)	2528			
1 to 3 years	6726	4365 (64.9)	2361	1824 (27.1)	378 (5.6)	159 (2.4)
3 to 5 years	5651	3728 (66.0)	1923	1506 (26.7)	301 (5.3)	116 (2.1)
5 to 10 years	4680	3092 (66.1)	1588	1248 (26.7)	250 (5.3)	90 (1.9)
10 to 15 years	1986	1252 (63.0)	734	592 (2938)	109 (5.5)	33 (1.7)

* Number of patients included in each study period

**Fig 1:**
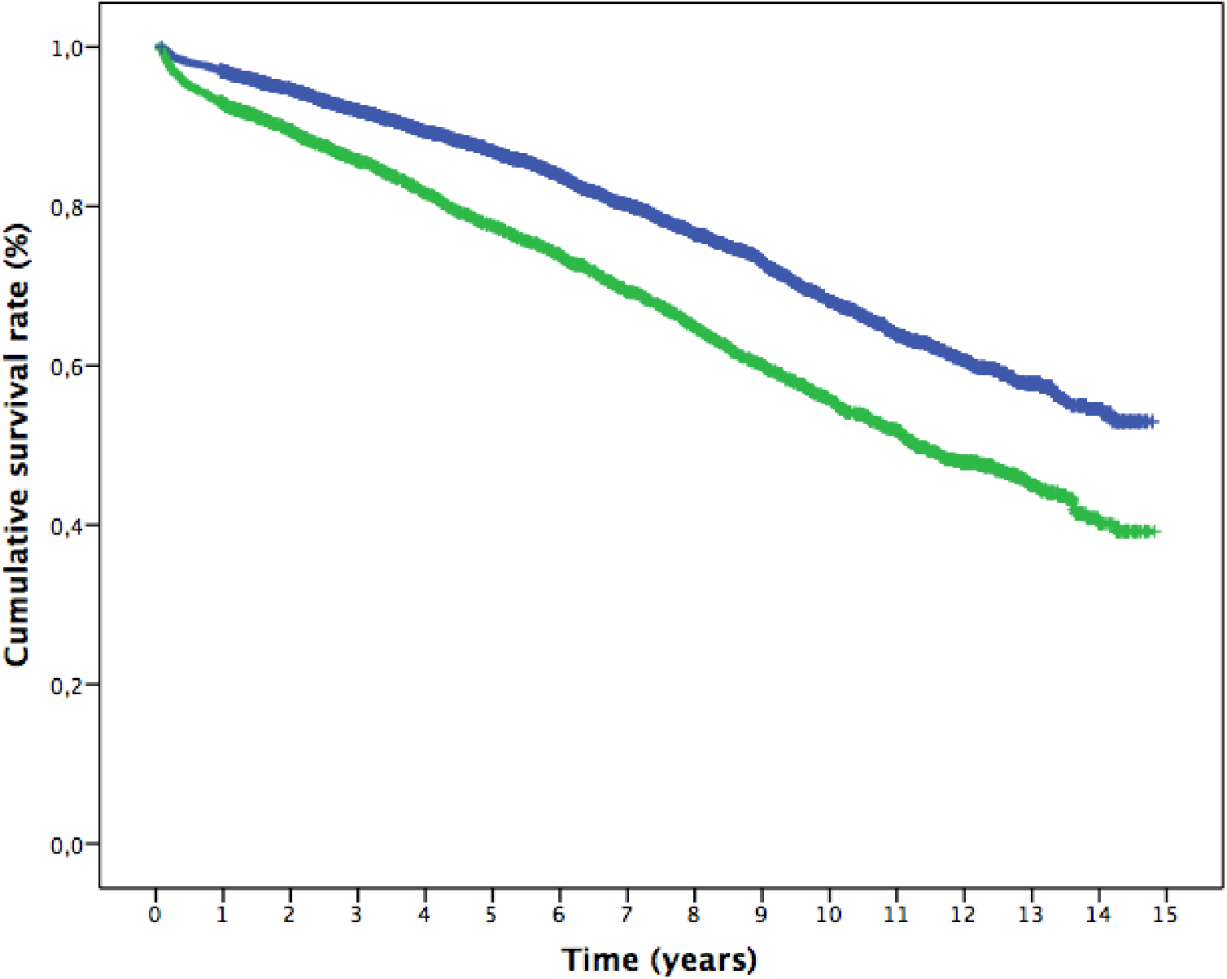
Kaplan-Meier cumulative survival curve from 30 days after surgery to 15 years follow-up. (Blue: patients without AKI, Green: patients who developed AKI). p<0.0001.

**Fig 2:**
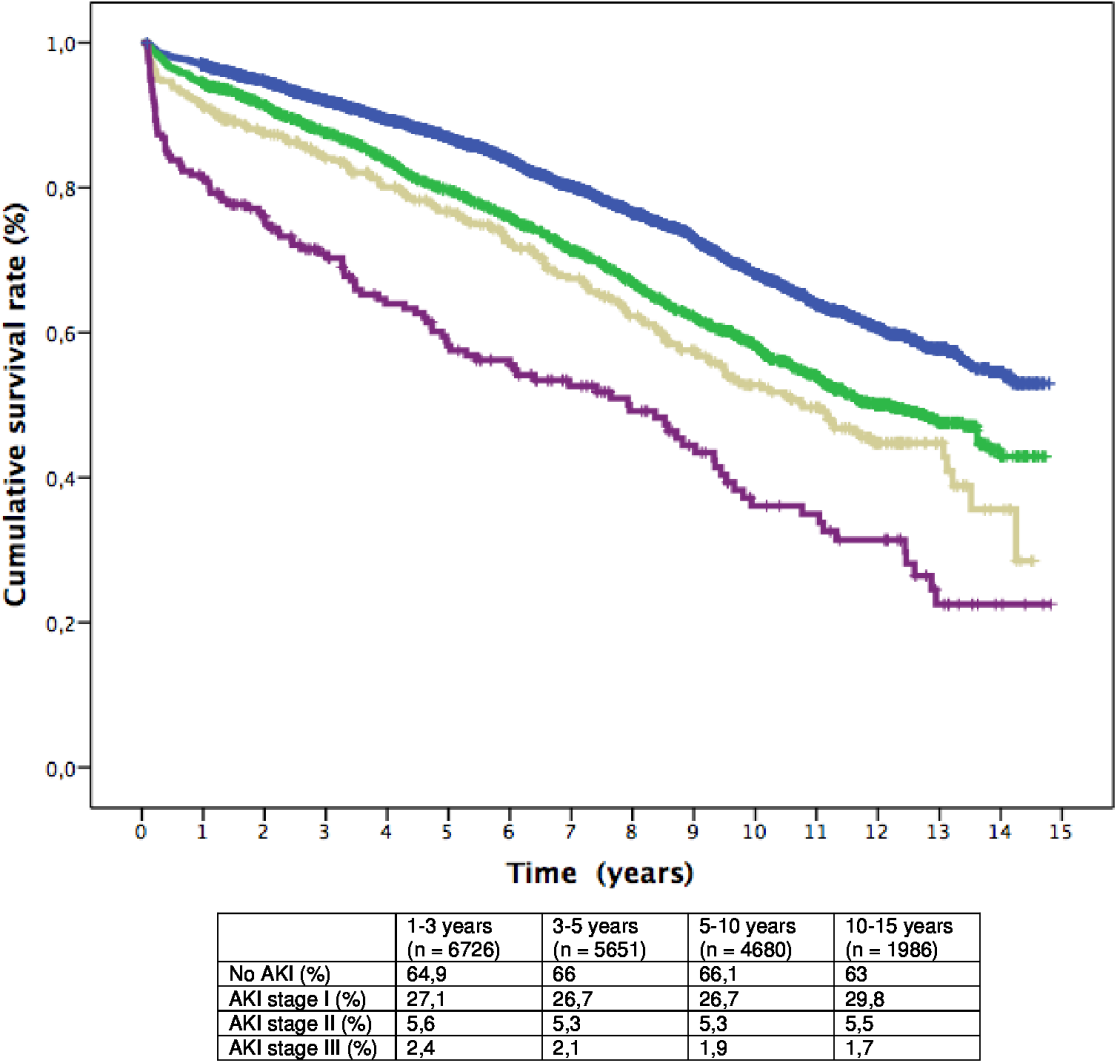
Kaplan-Meier cumulative survival curves from 30 days after surgery to 15 years follow-up. (Blue: patients without AKI; Green: patients who developed AKI stage I; Yellow: patients who developed AKI stage II; Purple: patients who developed AKI stage III); p< 0.0001. The table shows the percent distribution of patients in each period between groups (p = NS).

Probability of survival at 15 years for the full cohort of patients by AKI status and KDIGO stages was estimated using Kaplan-Meier curves. A lower long-term survival of all AKI patients over the follow-up period was found (survival AKI 39.2% SE 2.1% vs non-AKI 53% SE 1.8%, P = 0.000) and worsened with increased severity of AKI (Figs 1 and 2).

The Kaplan Meier cumulative survival curves showed a progressively decrease in the proportional difference in the slope of the curves between AKI categories, until all curves being parallel after 5 years follow-up (log-rank test, p = 0.08), then confirming a non-proportional risk distribution between AKI categories along time.

The results of the Cox proportional hazards model adjusted for comorbidities are presented in Table 3 in which are included only the associates variables to death and the period of follow-up in which occur. As shown, AKI was an independent predictor of death only during the first five years after surgery but was not significant during later follow-up intervals (30 days to 1 year: HR 1.834, 95% CI 1.459 to 2.306; 1 to 3 years: HR 1.285, 95% CI 1.023 to 1.610; and 3 to five years: HR 1.330, 95% CI 1.123 to 1.750). Only age, diabetes mellitus and CHF were associated with increased risk of death over the entire follow-up period. Other variables that were predictors of mortality in some but not all of the study periods were female sex, smoking, COPD, baseline renal function, peripheral vascular disease, cerebrovascular disease and previous myocardial infarction. Of note, the occurrence of stroke early after surgery remained associated with very late mortality.

**Table 3:** Cox proportional hazards model for co-morbidity adjusted impact of AKI on long-term mortality.

	30 d to 1 year HR (CI 95%)	1 to 3 years HR (CI 95%)	3 to 5 years HR (CI 95%)	5 to 10 years HR (CI 95%)	10 to 15 years HR (CI 95%)
Age	1.04 (1.02–1.05)	1.02 (1.01–1.04)	1.06 (1.05–1.07)	1.05 (1.04–1.06)	1.06 (1.05–1.08)
Female sex	0.85 (0.65–0.97)	0.75 (0.59–0.95)			
Diabetes mellitus	1.53 (1.21–1.93)	1.40 (1.12–1.74)	1.84 (1.45–2.06)	1.58 (1.36–1.83)	1.36 (1.02–1.81)
Smoker		1.30 (1.02–1.65)			
COPD	1.86 (1.33–2.61)	1.42 (1.02–1.98)	1.46 (1.03–2.06)	1.43 (1.12–1.82)	
eGFR	0.99 (0.99–1.00)	0.99 (0.98–0.99)		0.99 (0.99–1.00)	
PVD		1.63 (1.21–2.21)	1.72 (1.29–2.32)	1.26 (1.01–1.56)	
CVD	1.88 (1.13–3.12)				
Previous MI			1.36 (1.07–1.72)		
LVEF 30–50%	1.58 (1.25–1.98)	1.52 (1.22–1.89)	1.75 (1.41–2.18)	1.65 (1.44–1.89)	1.52 (1.18–1.95)
LVEF <30%	2.29 (1.36–3.85)	2.86 (1.83–4.46)	2.72 (1.53–4.82)	2.92 (2.00–4.26)	
Non CABG	1.29 (1.04–1.63)	1.86 (1.49–2.33)	1.73 (1.38–2.18)	1.33 (1.16–1.54)	
Post operatory stroke	4.53 (3.07–6.68)			2.34 (1.57–3.49)	3.07 (1.26–7.45)
Post operatory MI	3.64 (1.90–6.97)				
AKI	1.83 (1.46–2.31)	1.28 (1.02–1.61)	1.33 (1.12–1.75)		
KDIGO stage 1	1.49 (1.15–3.30)	1.93 (1.20–3.10)	2.26 (1.59–4.10)		
KDIGO stage 2	2.27 (1.56–3.30)		2.03 (1.25–3.30)		
KDIGO stage 3	4.36 (2.99–6.35)		2.13 (1.17–3.88)		

COPD indicates: chronic obstructive pulmonary disease; eGFR: estimated glomerular filtration rate; PVD: peripheral vascular disease; CVD: cerebrovascular disease; MI: myocardial infarction; LVEF: left ventricle excretion fraction; CABG: coronary artery by-pass grafting; AKI: acute kidney injury.

## Discussion

The most relevant finding of our study was the demonstration that AKI was a transient predictor of long-term mortality through until five years after surgery in a large cohort of patients with a very long-term follow-up time. Previous studies addressed to appraise the association of AKI with long-term outcomes in cardiac surgery [18–24] showed a uniformly reduced accumulative survival of AKI patients at the end of a follow-up time that ranged from 90 days to 10 years among studies. However, this changing prognostic role of AKI has received little attention to date, mainly in the middle follow-up time [18, 22]. Recently, Sawhney et al. [25] found a diminishing association of AKI with late mortality that was more evident after the first year of follow-up and in patients with lower baseline renal function in a large unselected population of 17630 patients. Neither of these studies reached follow-up time as long as 15 years.

We do not have an interpretation for our finding concerning the transient impact of an episode of AKI on long-term mortality. A simple explanation based on a dramatic reduction of surviving AKI patients at mid to long term follow-up can be ruled out since the proportion of patients with AKI remained unchanged throughout the entire 15 year period, as shown in Table 2. In addition, the possible effect of changes in the process of care of this patients could be ruled out since nor substantial modifications in the national health system neither major changes in surgical technique occurred in the study period.

The second valuable piece of information provided in the study relates to the changing pattern of the risk factor profile for death over time, as shown in the analysis of Kaplan-Meier curves (Fig 1) and in the Cox analysis in each period (Table 3) Non-modifiable variables such as age, cardiac failure, vascular events and diabetes remained associated with mortality along time. Previous studies also reported similar results as well as other independent predictors of late mortality such as COPD [20,24], baseline renal function [19,21], time on vasoactive drugs [21], length of hospital satay [20], duration of operation [19], procedural urgency [21,24] and preoperative atrial fibrillation [24]. The occurrence of stroke 30 days after surgery increased the risk of death fourfold during the first year, as expected. Notably, stroke reappeared as strong predictor of late mortality beyond five years. Underlying vascular damage addition to the delayed consequences of neurological sequelae and patient frailty could be a reasonable interpretation for this finding. Regarding AKI characteristics, similar to our study, all other studies demonstrated a decrease in survival rate with the severity of AKI, or the duration of AKI [26].

Finally, one methodological consideration should be put forward. The cumulative survival curve of all patients showed that the risk of death was non-constant along the study period, as the slope of the curve was more pronounced in the early years and then gradually tended to stabilize regardless the effect of aging on the expected mortality. Two relevant methodological caveats can be drawn from this finding. First, to the extent that the risk is not constant, the unrestricted use of the Cox proportional hazards model to explore the effects of several variables on survival is not appropriate. Second, under these circumstances, a Cox analysis stratifying the follow-up time at set intervals up to the end of the observation time as we did is more suitable given that risk prediction is highly dependent on the time point selected. The robustness of results will depend on the number of individuals at the beginning of each period in order to guarantee a statistical power able to identify differences in survival for each covariate included in the analysis. This dynamic point of view allows for the detection of changes in the relative burden imposed by covariates along time, particularly when non-modifiable variables such as gender and comorbidities coexist with unique postoperative events such as AKI, stroke or myocardial infarction.

Growing evidence presently leaves out of debate the association of a reversible or even mild episode of AKI with the risk of late adverse outcomes and premature death [5,12] in all settings in which AKI was studied. The challenge based on the current state of the knowledge is to elucidate the pathologic processes related to AKI and these untoward consequences and how the events associated with AKI relate to one other. Essentially, two theories have been proposed: AKI and post-AKI damage (fibrosis) are mediators of further renal and non-renal disease, or AKI is just a marker of an underlying adverse condition that results in undesirable results. To answer these questions, basic as well as clinical research designed specifically to explore the occurrence of changes in kidney structure and function over time is necessary. The new knowledge obtained will allow the design of strategies aimed to reduce the late burden associated with a reversible episode of AKI.

Our study has limitations. First, this is a retrospective analysis of a prospectively collected database. Second, the observational nature of the study could have residual bias and unknown confounding factors, as some components of the healthcare process as well as biological variability of patients potentially related to outcomes that were not reflected in our database. Third, even though this study was performed using a large, comprehensive and time-extended database, it was not designed for the purpose of the study. Fourth, 20.6% of patients received off-pump isolated CABG surgery, which was associated with a lower frequency of AKI (Table 1), that could bias the impact of AKI over long-term outcomes. Nevertheless, we were not able to demonstrate a net beneficial impact of this effect over long-term survival, in concordance with the results of the Coronary Study [27]. Fifth, according to the design of the follow-up questionnaire of our Institute, we were not able to detect the acquisition of new comorbidities or other relevant events that could affect late outcomes. Nevertheless, since the aim of the study was to evaluate the impact of an episode of AKI on long-term mortality adjusted to the clinical scenario at AKI occurrence this limitation is minimized. The strengths of this study include the large number of patients; the extent of information provided by the database covering the entire process of care until 30 days after surgery; the representativeness of the cardiac surgery field, since only aortic root surgery patients were excluded; the extension and completeness of follow-up as a series with the longest follow-up time published to date; and the appropriateness of the methodological tools and statistical analyses implemented.

## Conclusions

The present study demonstrates a transient association of a reversible episode of AKI with long-term mortality in a cardiac surgery setting. This association progressively decreases and vanishes beyond five years after surgery. The risk factor profile for late mortality was also dynamic, changing over time except for those clinical characteristics strongly related to the type of patients needing cardiac surgery such as advanced age, diabetes, cardiac failure and peripheral vascular disease. The concept that the relationship between AKI and long-term mortality is dynamic rather than static is crucial to understanding this complex and still poorly recognized association. We also highlight the importance of applying the most appropriate methodology to be able to identify the changing role of AKI as a prognostic marker at different follow-up times as well as the risk profile. By doing so, the post discharge management of these patients by a multidisciplinary team could be based on reliable clinical information rather than intuitive clinical judgment.
